# Elevated Seawater Temperatures Decrease Microbial Diversity in the Gut of *Mytilus coruscus*

**DOI:** 10.3389/fphys.2018.00839

**Published:** 2018-07-10

**Authors:** Yi-Feng Li, Na Yang, Xiao Liang, Asami Yoshida, Kiyoshi Osatomi, Deborah Power, Frederico M. Batista, Jin-Long Yang

**Affiliations:** ^1^International Research Center for Marine Biosciences, Ministry of Science and Technology, Shanghai Ocean University, Shanghai, China; ^2^Key Laboratory of Exploration and Utilization of Aquatic Genetic Resources, Ministry of Education, Shanghai Ocean University, Shanghai, China; ^3^National Demonstration Center for Experimental Fisheries Science Education, Shanghai Ocean University, Shanghai, China; ^4^Graduate School of Fisheries and Environmental Sciences, Nagasaki University, Nagasaki, Japan; ^5^Centro de Ciencias do Mar (CCMAR), Universidade do Algarve, Campus de Gambelas, Faro, Portugal; ^6^Department of Biosciences, College of Science, Swansea University, Swansea, United Kingdom

**Keywords:** elevated seawater temperature, gut microbiome, Illumina MiSeq sequencing, *Mytilus coruscus*, 16S rRNA

## Abstract

The gut microbial community is critical for the host immune system, and in recent years, it has been extensively studied in vertebrates using ‘omic’ technologies. In contrast, knowledge about how the interactions between water temperature and diet affect the gut microbiota of marine invertebrates that do not thermoregulate is much less studied. In the present study, the effect of elevated seawater temperature and diet (*Isochrysis zhanjiangensis* and *Platymonas helgolandica* var. *tsingtaoensis*) on the gut microbial community of the commercial mussel, *Mytilus coruscus*, was investigated. The 16S rRNA gene sequencing was used to characterize the microbial community in *M. coruscus* gut. The mortality of *M. coruscus* exposed to a high water temperature (31°C) increased after 3 days and the diversity of the bacterial community in the gut of live *M. coruscus* was significantly reduced. For example, the abundance of *Bacteroides* (Bacteroidetes) and norank_*Marinilabiaceae* (Bacteroidetes) increased in the gut of *M. coruscus* fed *I. zhanjiangensis*. In *M. coruscus* fed *P. helgolandica*, the abundance of *Arcobacter* (Proteobacteria) and norank_*Marinilabiaceae* increased and the abundance of unclassified_*Flavobacteriaceae* (Bacteroidetes) decreased. The results obtained in the present study suggest that high temperatures favored the proliferation of opportunistic bacteria, including *Bacteroides* and *Arcobacter*, which may increase host susceptibility to disease. Microbial community composition of the gut in live *M. coruscus* was not impacted by the microalgal diet but it was modified in the group of mussels that died. The present study provides insight into the potential effects on the gut microbiome and mussel–bacteria interactions of rising seawater temperatures.

## Introduction

The composition of the community of microorganisms (microbiota) that colonize the body surface of animals is driven by environmental factors and can have a crucial impact on metabolism, survival, homeostasis, and development of the host (Tremaroli and Bäckhed, 2012; McFall-Ngai et al., 2013). An important role of the host’s commensal microbiota is to protect it from pathogens by inhibiting their overgrowth through niche competition (Kamada et al., 2013), although sometimes commensal bacteria can become pathogenic (Garnier et al., 2007; Olson et al., 2014). Understanding how environmental factors affect host-microbiota interactions are important for aquaculture since they can be used to control and mitigate the impact of infectious diseases and hence improve animal health and survival.

Aquatic animals are in a medium that favors exposure to microbes because the diet, water, and sediments all harbor high levels of microorganisms that can colonize the body surfaces. The gut microbiota of shellfish is proposed to be dependent on the water’s microbial content due to the large volume of water that flows through this tissue (Gatesoupe, 1999). In abalone (*Haliotis midae*), the diet can modulate the polysaccharide degrading bacteria in the gut and contribute to digestion (Erasmus et al., 1997). In fact, probiotics, live microbial feed supplementation, are proposed to benefit the health of the host, and they are frequently used in larval fish and shellfish aquaculture (Gatesoupe, 1999). Additional beneficial effects proposed for the microbiota in aquatic animals are increased disease resistance due to their stimulatory effect on the immune system, and production of nutrients (Marques et al., 2006). Understanding host-microbiota interactions, therefore, can give insight into the likely consequences of rising seawater temperatures and provide alternative approaches for improving health and mitigating disease in the aquaculture industry.

Global climate change is affecting microbial diversity, function, and community dynamics in marine ecosystems (Webster and Hill, 2007), and as a consequence host-microbe interactions is also shifting. A rise in seawater temperatures may favor the shift from beneficial bacteria to pathogen-dominated microbes and increase the susceptibility of the host to diseases (Lafferty et al., 2004; Ritchie, 2006). In the case of the Pacific oyster (*Crassostrea gigas*), the composition of the hemolymph microbiota was affected by seawater temperatures (Lokmer and Wegner, 2015). The microbiome was also modified in the digestive gland of the Sydney rock oyster (*Saccostrea glomerata*) infected with a paramyxean parasite and the gut of the abalone (*Haliotis discus* hannai) fed a modified diet (Tanaka et al., 2003; Green and Barnes, 2010). These studies reveal that numerous factors can modify the microbiome in bivalves and highlight the need for studies that look at several parameters simultaneously.

The impact of climate change on bivalve mortality is of high interest for the aquaculture industry. Summer Mortality Syndrome (SMS) affects bivalves, occurs regularly in summer, and has a highly negative impact on aquaculture worldwide. Mass mortality events linked to SMS have been reported in marine invertebrates such as the blue mussel (*Mytilus edulis*), *C. gigas*, the zhikong scallop (*Chlamys farreri*), and the European abalone (*Haliotis tuberculata*) (Myrand et al., 2000; Xiao et al., 2005; Travers et al., 2008; Malham et al., 2009). Although the causal factors of SMS are unclear, increasing seawater temperature reduced the immune response and resistance to infectious diseases and in China since 1998 have led to mass mortalities of the abalone (*Haliotis diversicolor supertexta*) due to vibriosis outbreaks (Cheng et al., 2004). The hard-shelled mussel, *Mytilus coruscus*, is a commercially important bivalve in China, which can be found from the Bohai Sea to the South China Sea. Mass mortality of *M. coruscus* in aquaculture has been associated with viral outbreaks, which in 1995 caused 70% mortality (Zhang et al., 1999). The sporadic nature of pathogen associated mortality events in mussels, the likely positive influence of the commensal microbiota, and the broad geographic range and temperature tolerance make them an interesting model for studies of the impact of host-microbiota interactions.

In the present study, the combined effects of temperature and diet on the gut microbial community of *M. coruscus*, an important aquaculture bivalve in China, were determined using Illumina MiSeq sequencing of 16S rRNA. The relative importance of seawater temperature and diet (two different microalgae were supplied) on the microbiota structure and diversity in the mussel gut was investigated. The likely impact of the changes in microbiota structure on host susceptibility was assessed by determining the change in the relative size of the population of opportunistic bacteria.

## Materials and Methods

### Ethics Statement

The experimental protocol for mussel acclimation and experimentation was approved by the Animal Ethics Committee of Shanghai Ocean University, Shanghai, China.

### Biological Material

Adult *M. coruscus* mussels were collected at Gouqi Island, (30°72′N; 122°77′E), Zhoushan, Zhejiang Province, China, in August 2016. Upon arrival in the laboratory, the mussels were immediately cleaned and kept in 10-l polycarbonate tanks (∼30 mussels/tank) containing seawater (salinity: 30 ppt) at 27°C, which is the average temperature in the site where the mussels were collected during the summer season. All mussels were fed daily with *Platymonas helgolandica* var. *tsingtaoensis* and *Isochrysis zhanjiangensis*, and the algal density in the tank water was maintained at 2 × 10^3^ cells⋅ml^-1^ and 1 × 10^4^ cells⋅ml^-1^, respectively. *P. helgolandica* and *I. zhanjiangensis* were chosen due to their tolerance and high growth rate under high temperatures relative to other species (Wang and Wang, 1995). The seawater was changed every other day using concentrated seawater from the same location as the mussels (Zhoushan, China) diluted to the appropriate concentration with clean aerated tap water. Mussels were acclimated to the experimental tanks for 1 week before the start of the experiment. The shell length and width of the mussels used for the experiment were 8.8 ± 0.3 cm and 4.3 ± 0.3 cm, respectively.

### Experimental Setup and Gut Sampling

Mussels were exposed for 3 days to the combined effects of temperature and different diets, and the changes in the gut microbiota were determined. The mussels (*n* = 45) were kept in triplicate 10-l polycarbonate tanks at 27°C ± 1°C (Control) or 31°C ± 1°C and fed daily with *I. zhanjiangensis* or *P. helgolandica* (algal cell density of 4 × 10^2^ cells⋅ml^-1^). The temperature challenge administered was 31°C, the upper limit for survival of *M. coruscus* mussels and this temperature corresponds to the predicted rise in temperature by 2100 (assuming an overall global air temperature increase of 1.8–4°C, IPCC, 2007). For the treatments at 31°C, the temperature was gradually increased (0.15°C⋅h^-1^) to minimize heat shock (Webster et al., 2011).

The experiment was composed of three main treatment groups in triplicate (15 mussels per tank): the baseline (day 0, maintained at 27°C and fed with *I. zhanjiangensis* and *P. helgolandica*), control (maintained at 27°C and fed with *I. zhanjiangensis* or *P. helgolandica* for 3 days), and treatment (maintained at 31°C and fed with *I. zhanjiangensis* or *P. helgolandica* for 3 days) (**Table 1**). The baseline groups were sampled at day 0 of the trial (3 individuals per replicate tank) to assess the microbiota at the beginning of the experiment. In the remaining groups, the tanks were checked frequently (at least 3 times a day) and dead mussels were immediately removed when identified and the cumulative mortality was registered on a 24-h basis. After 3 days of exposure to the experimental conditions, gut tissues from live mussels and mussels that had recently died (without signs of decomposition) were excised aseptically using sterile scissors and tweezers. Gut tissues were placed in 1.5 ml sterile tubes and immediately frozen at -80°C. Samples of *I. zhanjiangensis* and *P. helgolandica* were also collected for analysis to assess whether the microalgae microbiota affected the mussel gut microbiome.

**Table 1:** The treatment condition of the mussel *M. coruscus*.

Assays	Temperature	Diet	Sample time (day)	Mussel condition	
Baseline	27°C	*I. zhanjiangensis* and *P. helgolandica*	0	live	–
Control	27°C	*I. zhanjiangensis*	3	live	dead
	27°C	*P. helgolandica*	3	live	dead
Treatment	31°C	*I. zhanjiangensis*	3	live	dead
	31°C	*P. helgolandica*	3	live	dead

### DNA Extraction and PCR Amplification

The defrosted gut samples were initially ground in extraction buffer with a hand held pestle and mortar and samples were efficiently lysed by adding a mixture of ceramic and silica particles. Total DNA was extracted (*n* = 3) using a FastDNA^TM^ Spin Kit for Soil (MP Bio, United States) and following the protocol provided by manufacturer. MT Buffer and Sodium Phosphate Buffer from the kit were used for tissue homogenization. DNA concentration and purity were analyzed using a Nanodrop 2000 (Thermo Scientific) and the DNA content (5 ng⋅μl^-1^) of each sample was standardized by adjusting the final volume with sterile, filtered (0.2 μm) water.

The 16S ribosomal RNA gene (16S rRNA, V3-V4 region) from bacteria was amplified using previously reported universal bacterial primers, 338F (5′-ACTCCTACGGGAGGCAGCAG-3′) and 806R (5′-GGACTACHVGGGTWTCTAAT-3′) (Caporaso et al., 2010). Primers were tagged with a unique barcode for each sample prior to sequencing. A total of 20 microliter PCR reactions were performed as follows: 4 μl of 5 × FastPfu Buffer, 2 μl of 2.5 mM dNTPs, 0.8 μl of forward primer (5 μM), 0.8 μl of reverse primer (5 μM), 0.4 μl of FastPfu Polymerase, 0.2 μl of BSA, 10 ng of template DNA, and ddH_2_O. The PCR thermocycle used for 16S rRNA amplification was: 3 min denaturation at 95°C; 27 cycles of 30 s at 95°C, 30 s at 55°C, 45 s at 72°C; and a final elongation step of 10 min at 72°C.

### Illumina MiSeq Sequencing

The PCR products were checked on 2% agarose gels, the target bands were extracted, then purified using an AxyPrep DNA Gel Extraction Kit (Axygen Biosciences, United States) and quantified using a QuantiFluor^TM^-ST (Promega, United States). Three cDNA libraries that each corresponded to a pool of DNA extracted individually from three different individuals were used for sequencing. Primers for each cDNA library had a specific barcode, which was used to distinguish the different samples. Equimolar concentrations of the purified amplicons from each cDNA library were pooled, and sequencing (2 × 250 bp) was performed using an Illumina MiSeq platform (Majorbio, Shanghai, China). The raw sequences have been submitted to the NCBI sequence read archive database under the accession number: SRP 120925.

### Statistical and Bioinformatics Analysis

Mortality data for *M. coruscus* and the percentages of the relative abundance of bacterial phyla, family, and genus were arcsine-square root transformed. All data were tested for normality (Shapiro–Wilk test) and homogeneity (O’Brien test). Mortality rate, the percentage of the relative abundance of bacterial species, and microbial diversity indices (Chao1, Shannon, and Simpson) were analyzed using a Kruskal–Wallis test followed by a Steel-Dwass All Pairs test. Similarity of percentage (SIMPER) analysis was used to compare the major bacterial species contributing to the dissimilarity between the control and the treatment groups using Primer 6 software (Primer-E Ltd.). The data were analyzed using JMP^TM^ software (SAS Institute, Shanghai, China). Results with a *P*-value < 0.05 were considered significantly different.

QIIME was used to analyze raw fastq data (Caporaso et al., 2010). The raw paired-reads fastq files were demultiplexed and quality-filtered. The Usearch pipeline^1^ (version 7.0) was used to determine the OTUs using a similarity threshold of 97%. The taxonomic affiliation of each 16S rRNA sequence was determined with the RDP classifier^2^ against the Silva database^3^ (Release128) using a confidence threshold of 70%. Principal Component Analysis (PCA) was performed to explore the structure of the bacterial communities using the cloud platform of I-Sanger^4^. The richness index of Chao1 and the Simpson’s and Shannon diversity index were calculated using R.

## Results

### *M. coruscus* Mortality

**FIGURE 1:**
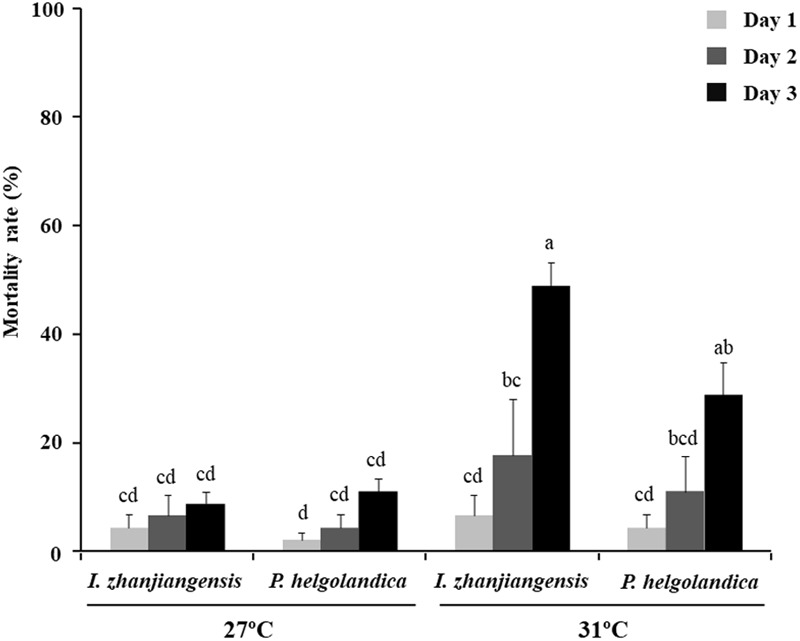
Mortality rates for *M. coruscus*. Data are the mean ± SE (*n* = 3). Different letters indicate significant differences (*P* < 0.05).

The mortality rates of mussels cultured at 27°C and 31°C and fed two different microalgae diets for 3 days are shown in **Figure 1**. After 3 days, the mortality rates were significantly higher in the mussels kept at 31°C relative to those at 27°C (*P* < 0.05). The highest mortality rate (48.9% ± 4.4%) was observed in the mussels fed with *I. zhanjiangensis* and maintained at 31°C, although this was not significantly different from those maintained at 31°C and fed *P. helgolandica* (*P* > 0.05).

### Gut Microbial Community Analysis Based on MiSeq Sequencing

Globally 1420 operational taxonomic units (OTUs) were identified from the gut samples collected from mussel maintained at 27°C or 31°C and fed one or both of the microalgae diets. When a 3% dissimilarity level was used to analyze the gut samples, Good’s coverage showed that 99.3–99.9% of the OTUs were identified in all of the groups analyzed.

### Microbiota Phylum Detected in the Mussel Gut

**FIGURE 2:**
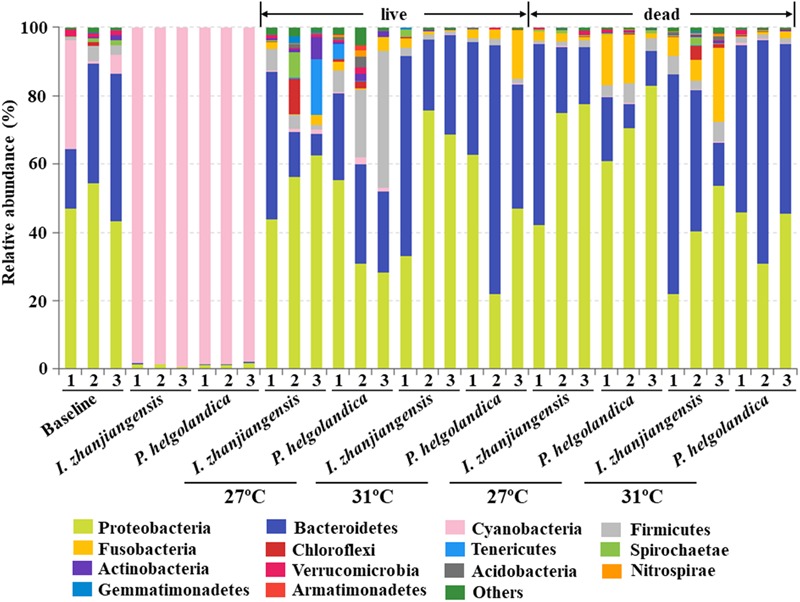
Relative abundance of bacterial communities at the phylum level of gut and microalgae samples. Three replicates are labeled with the numbers 1, 2, and 3.

A total of 14 different phyla with an abundance > 1% were identified, and the abundance of 27 phyla < 1% were all classified as “others” (**Figure 2**). Significant differences between mussel groups existed in the relative abundance of 14 phyla except Armatimonadetes (*P* < 0.05, **Figure 2**, Supplementary Table S1). In the rare phyla, there was significant difference in the relative abundance of Chlorobi, Planctomycetes, SBR1093, WS6, Fibrobacteres, Deinococcus-Thermus, Saccharibacteria, and TM6_Dependentiae (*P* < 0.05, Supplementary Table S1) between the control and treatment groups. In the mussel fed one of the two microalgae diets, Cyanobacteria was the dominant phyla (> 97% of all sequences) and no significant difference was observed in the relative proportion of Cyanobacteria between these two groups (*P* > 0.05, **Figure 2**, Supplementary Table S1). The majority of the reads (65–99% of total OTUs) obtained from the mussel gut were assigned to Proteobacteria, Bacteroidetes, and Firmicutes (**Figure 2**, Supplementary Table S1). The two microalgae diets had no effect on the abundance of Proteobacteria, Bacteroidetes, and Firmicutes except in the mussels that died (*P* > 0.05, Supplementary Table S2). In the live mussels, an increase in the water temperature caused a decrease in the relative abundance of Firmicutes, when they had been fed with *P. helgolandica* (*P* < 0.05, Supplementary Table S2). In the dead mussel groups, all three bacterial phyla (Proteobacteria, Bacteroidetes, and Firmicutes) were significantly changed in the mussel groups fed with *P. helgolandica* irrespective of temperature (*P* < 0.05, Supplementary Table S2).

### Microbiota Families Detected in the Mussel Gut

**FIGURE 3:**
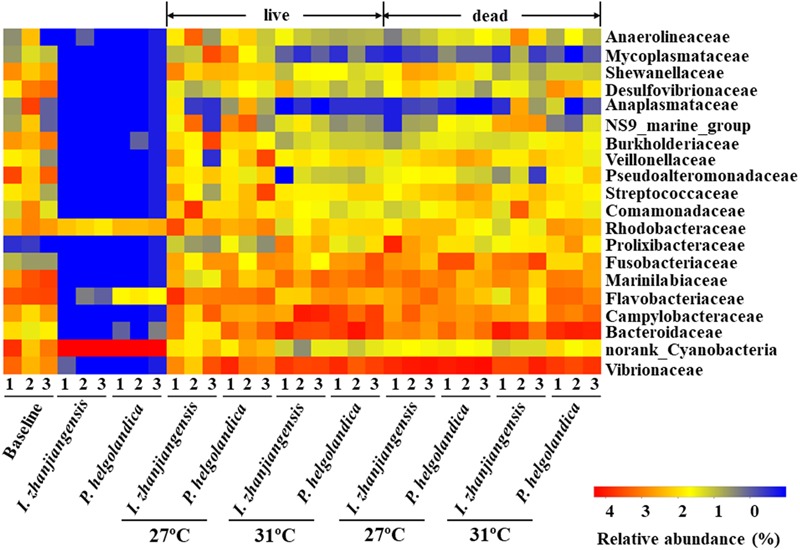
Heatmap revealing the top 20 bacterial families (%) of gut and microalgae samples. Three replicates are labeled with the numbers 1, 2, and 3.

The 20 most abundant families in mussel gut were selected for comparative analysis (**Figure 3**). Vibrionaceae, Bacteroidaceae, Campylobacteraceae, Flavobacteriaceae, and Marinilabiaceae were the five dominant families present in the control and treatment groups (5–93% of total reads, **Figure 3**, Supplementary Table S3). In mussels fed one of the two microalgae diets no change in the relative abundance of Vibrionaceae, Bacteroidaceae, Campylobacteraceae, Flavobacteriaceae, and Marinilabiaceae was identified except in the gut of mussels maintained at 31°C that had recently died (*P* > 0.05, Supplementary Table S4). In the live mussel groups, a higher water temperature (31°C) caused a significant increase in the relative abundance of Bacteroidaceae and Marinilabiaceae when they were fed with *I. zhanjiangensis* (*P* < 0.05, Supplementary Table S4). In contrast, a significant increase in the abundance of Campylobacteraceae and Marinilabiaceae occurred in the live mussels that were fed with *P. helgolandica*, and this was accompanied by a significant decrease in the relative abundance of Flavobacteriaceae (*P* < 0.05, Supplementary Table S4). In the recently dead mussel groups, a variation in the relative abundance of Vibrionaceae, Bacteroidaceae, and Flavobacteriaceae was only observed when the mussels had been fed with *P. helgolandica* prior to death (*P* < 0.05, Supplementary Table S4).

### Microbiota Genus Detected in the Mussel Gut

**FIGURE 4:**
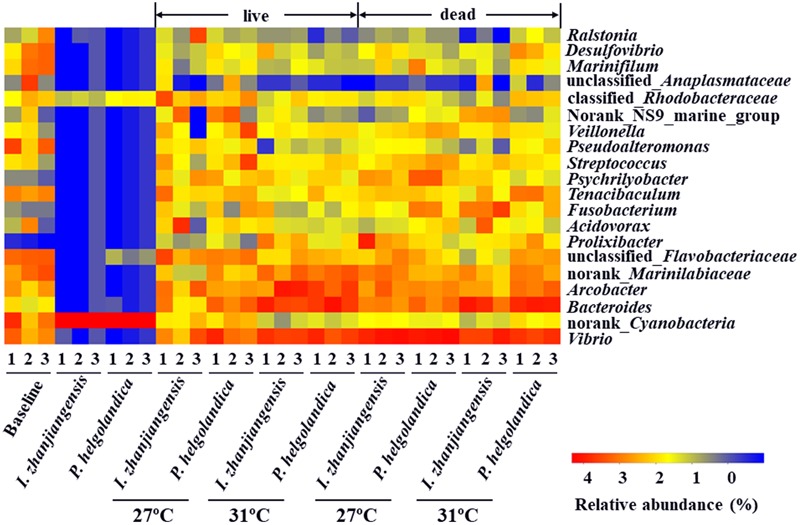
Heatmap revealing the top 20 bacterial genera (%) of gut and microalgae samples. Three replicates are labeled with the numbers 1, 2, and 3.

A heatmap of the 20 most abundant genera was constructed for comparative analysis (**Figure 4**). The top five bacterial genera identified in the control and treatment groups included *Vibrio, Bacteroides, Arcobacter*, norank_*Marinilabiaceae*, and unclassified_*Flavobacteriaceae* (3–93% of total reads, **Figure 4**, Supplementary Table S5). The two microalgae diets did not significantly modify the relative abundance of the five top genera in the live mussel groups (*P* > 0.05, Supplementary Table S6). Exposure of mussels to a higher water temperature (31°C) was correlated with a significant increase in the abundance of *Bacteroides* and norank_*Marinilabiaceae* in the live mussel groups fed with *I. zhanjiangensis* (*P* < 0.05, Supplementary Table S6). SIMPER analysis indicated that the genera *Bacteroides* and norank_*Marinilabiaceae* accounted for the dissimilarity of 0.95 and 0.69% in the live mussel groups fed with *I. zhanjiangensis* (Supplementary Table S7). The relative abundance of *Arcobacter* and norank_*Marinilabiaceae* significantly increased in the live mussels fed with *P. helgolandica* and contributed to > 0.5% of the variance (*P* < 0.05, Supplementary Tables S6, S7).

**FIGURE 5:**
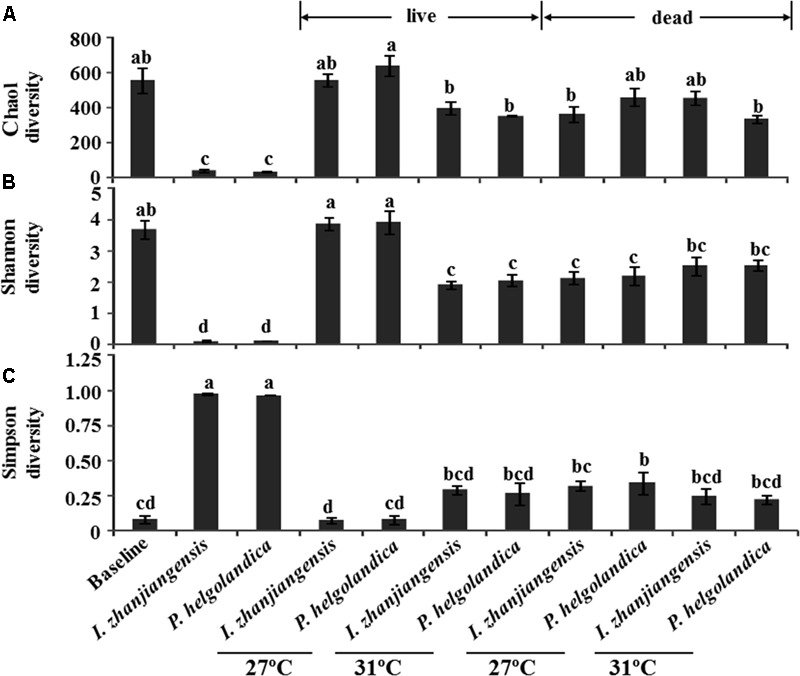
Microbial diversity indices of Chao **(A)**, Shannon **(B)**, and Simpson **(C)**. Data are the mean ± SE (*n* = 3). Different letters mean significant differences (*P* < 0.05).

The total species abundance index of Chao1 (Chao, 1984) and the species diversity indices of Shannon or Simpson are shown in **Figure 5**. No significant difference in the Chao1 index was identified in the mussel maintained at 27°C and fed both *I. zhanjiangensis* and *P. helgolandica* (the baseline group) relative to the groups fed either *I. zhanjiangensis* and *P. helgolandica* and maintained at 27°C (control) or at 31°C (treatment, *P* > 0.05). The lowest Chao1 index was observed for the microbiota of the two microalgae. A higher water temperature of 31°C caused a significant reduction in the Chao1 index of the live mussels fed with *P. helgolandica* relative to those maintained at 27°C (*P* < 0.05). Similarly, a significant decrease in diversity (*P* < 0.05) was indicated by the Shannon index in the live mussels fed either *P. helgolandica* or *I. zhanjiangensis* and exposed to higher water temperatures (31°C) relative to those maintained at 27°C. No significant differences in the Shannon index were found between the live mussels relative to the dead mussels at 27°C or 31°C irrespective of diet. The Simpson Diversity Index was not significantly different between the baseline group and any other the other groups within the experiment, with the exception of the dead mussels collected from the group maintained at 27°C and fed *P. helgolandica* when they were alive (*P* < 0.05).

**FIGURE 6:**
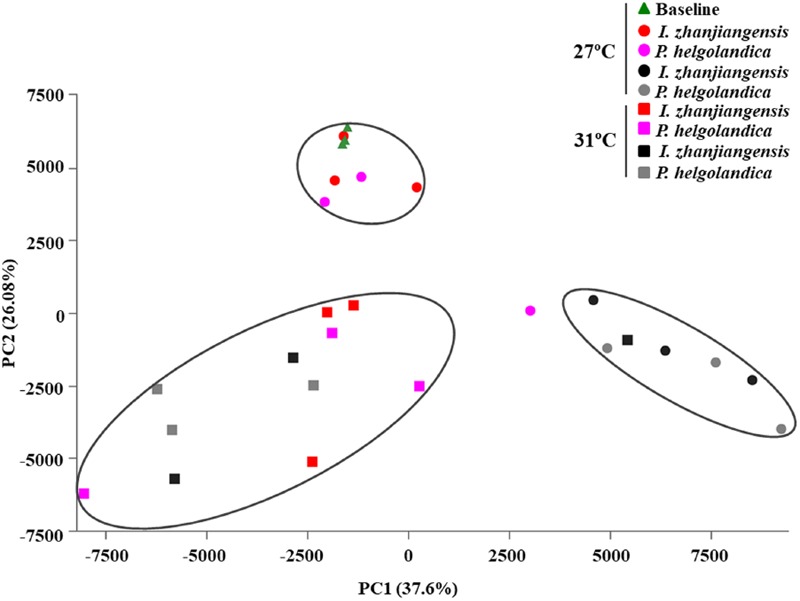
Principal components analysis of gut microbiome. Red and pink spot or square means live mussel groups. Black and gray spot or square means dead mussel groups; the triangle and spot groups mean the mussels cultured at 27°C; the square groups mean the mussels cultured at 31°C.

The PCA revealed that a seawater temperature of 31°C altered the bacterial communities in the gut of *M. coruscus* when compared to *M. coruscus* maintained at 27°C (**Figure 6**). At 27°C, clear differences were observed in the bacterial communities between the live/dead mussel groups. However, the same tendency was not observed for the community composition of bacteria in the gut samples from live and dead mussels at 31°C. Overall, the two microalgal diets had less effect on the bacterial communities than temperature.

## Discussion

The present study showed, as expected, an increase in mussel mortality when the sea water temperature was raised from 27 to 31°C. The increased mortality was associated with a decrease in the gut microbial diversity as assessed by 16S rRNA sequencing in both dead and live mussels at 31°C. PCA analysis suggested that an increase in seawater temperature from 27 to 31°C affected the bacterial community composition in the gut of the live mussels. SIMPER analysis revealed that the genus *Bacteroides* contributed to the highest dissimilarity between microbial communities in live mussels fed with *I. zhanjiangensis* and *Arcobacter* contributed to the highest dissimilarity in live mussels fed with *P. helgolandica* when considering the top five bacterial genera.

Previous studies have shown that the microbial community varied in the gut of the model vertebrate zebrafish across its development, and also indicated that inter-individual differences in the microbiome may be a characteristic of vertebrates during their development (Stephens et al., 2016). In the case of the invertebrates, the gut microflora of the abalone, *H. discus*, altered with the change in diet characteristic of this period of the developmental program (Tanaka et al., 2003). Similarly, the microbial community in the intestine of the sea bass, *Dicentrarchus labrax*, was modified between fish fed two different diets for four weeks, indicating that diet can significantly impact the gut microbiota in this fish (Carda-Diéguez et al., 2013). The results of our study of the gut microbiota in mussels fed different microalgae for 3 days revealed that diet did not significantly change the microbiota at the level of the phylum, family, and genera. The discrepancy observed in relation to the effect of diet on the gut microbiome in our study relative to previous studies may be due to species differences but also the relatively short period of the experimental trial.

Warm temperatures can lead to heterogeneity of bacterial community composition (Erwin et al., 2012). In the present study, the results of the PCA analysis showed that the gut bacterial communities in the live mussels at 27°C and 31°C were clearly separated, which suggests that the major bacterial species were strongly influenced by temperature. A similar effect of temperature has been reported in *C. gigas* and the hemolymph microbial dynamics and communities were modified by an increase in water temperature that also caused a high mortality rate (Lokmer and Wegner, 2015). However, it is noteworthy in the present study that the microbiome in the gut of live and dead mussels at 31°C was similar, as indicated by PCA analysis (**Figure 6**). Thus, the lower microbial diversity and shift in the gut microbiota of the live mussels at 31°C relative to the mussels at 27°C may have been a contributing factor in the resulting mortality. It remains to be established whether the shift in the bacterial community was due to a change in the mussel’s physiology caused by the increased water temperature or a direct response of the bacteria to temperature.

A drop in the microbial diversity of the gut in mussels exposed to increased water temperatures (31°C) was revealed by the Shannon and Simpson indexes and showed that temperature had a more significant impact on diversity of the gut microbiota than diet in this study. Low microbial diversity has been reported in unhealthy oysters under heat stress (Lokmer and Wegner, 2015). It has been proposed that species-rich microbiota in healthy animals increases their resilience under adverse conditions and promotes a high resilience threshold (Lozupone et al., 2012). This is indirectly supported by studies that reveal impaired health status is strongly associated with low microbial diversity in many organisms (Garnier et al., 2007; Chang et al., 2008; Green and Barnes, 2010). The effect of low microbial community diversity on community interactions and function is complex, but it has recently been proposed that there are key species of bacteria that are most important in defining microbiota function in the host gut (Marchesi et al., 2016).

The present study revealed that the mussel gut was dominated by three bacterial phyla, and these dominant bacteria presumably play an important role in gut function. Firmicutes are often found in the gut of marine invertebrates such as sea squirt (*Ciona intestinalis*) (Dishaw et al., 2014), abalone (*Haliotis diversicolor*) (Zhao et al., 2012), eastern oyster (*Crassostrea virginica*) (King et al., 2012), black tiger shrimp (*Penaeus monodon*) (Rungrassamee et al., 2014), Atlantic blue crab (*Callinectes sapidus*) (Givens et al., 2013), and the sea urchin (*Lytechinus variegatus*) (Hakim et al., 2015). Our study revealed that high water temperatures reduced the relative abundance of Firmicutes in the gut of live mussels fed with the microalga, *P. helgolandica*. This observation is consistent with a previous study, which showed a reduction in Firmicutes abundance in the gut of unhealthy crabs (*Eriocheir sinensis*) (Ding et al., 2017).

An increase in water temperature (from 27 to 31°C) had no influence on the abundance of Proteobacteria and Bacteroidetes at the phylum level, but increased the relative abundance of Bacteroidaceae and Marinilabiaceae which belong to the phylum Bacteroidetes in live mussels fed with *I. zhanjiangensis* (Supplementary Tables S2, S4). In the gut of the mussels exposed to 31°C, there was a high relative abundance of the genus *Bacteroides* in the family Bacteroidaceae (Supplementary Table S6). Previous reports in crab have indicated that the *Bacteroides* species may include opportunistic pathogens associated with disease and their presence contributed to morbidity and mortality (Redondo et al., 1995; Liu et al., 2003; Li K. et al., 2007; Li Y. et al., 2007; Patrick et al., 2009). *Arcobacter* species are common in many marine invertebrates, such as crabs (Givens et al., 2013), mussels (Collado et al., 2009), abalones (Tanaka et al., 2004), and oysters (Romero et al., 2002). Moribund oysters with low microbial diversity had a high abundance of *Arcobacter* species and this species has been proposed as an indicator of impaired animal health (Lokmer and Wegner, 2015). In the present study, the *Arcobacter* species contributed highly in the live groups fed with *P. helgolandica* and in the dead groups fed either of the two diets (Supplementary Table S6), indicating that the *Arcobacter* may contain species that are opportunistic pathogens when present in high densities and it is possible that they contributed to mussel death.

The results obtained in the present study support the hypothesis that an increase in water temperature may change the gut microbiota in mussel and favor the proliferation of opportunistic bacteria. In addition, the microalgal diets used in the present study (*P. helgolandica* and *I. zhanjiangensis*) did not significantly influence the gut microbial community. Better knowledge about the factors that influence host microbial communities in invertebrates are required for the development of strategies to promote animal health and prevent or mitigate the impact of infectious diseases in the shellfish aquaculture industry.

## Author Contributions

J-LY and XL conceived and designed the experiments. NY and Y-FL performed the experiments. Y-FL and J-LY analyzed the data. Y-FL, FB, DP, and J-LY critically reviewed the data. Y-FL, FB, DP, J-LY, AY and KO wrote the manuscript. All authors reviewed the manuscript.

## Conflict of Interest Statement

The authors declare that the research was conducted in the absence of any commercial or financial relationships that could be construed as a potential conflict of interest.

## Footnotes

1 http://drive5.com/uparse/

2 http://rdp.cme.msu.edu/

3 http://www.arb-silva.de

4 http://www.i-sanger.com/
